# Functional Status Following Pulmonary Rehabilitation: Responders and Non-Responders

**DOI:** 10.3390/jcm11030518

**Published:** 2022-01-20

**Authors:** Sara Souto-Miranda, Maria A. Mendes, João Cravo, Lília Andrade, Martijn A. Spruit, Alda Marques

**Affiliations:** 1 Lab3R-Respiratory Research and Rehabilitation Laboratory, and Institute of Biomedicine (iBiMED), School of Health Sciences (ESSUA), University of Aveiro, 3810-193 Aveiro, Portugal; sara.souto@ua.pt; 2 Department of Medical Sciences, University of Aveiro, 3810-193 Aveiro, Portugal; 3 Department of Respiratory Medicine, Maastricht University Medical Centre, NUTRIM School of Nutrition and Translational Research in Metabolism, Faculty of Health, Medicine and Life Sciences, Maastricht University, 6200 MD Maastricht, The Netherlands; martijnspruit@ciro-horn.nl; 4 Department of Pulmonology, Centro Hospitalar do Baixo Vouga, E.P.E., 3810-164 Aveiro, Portugal; mamendes88@gmail.com (M.A.M.); joaocravopt@hotmail.com (J.C.); m.lilia.andrade@hotmail.com (L.A.); 5 Department of Research and Development, Ciro, 6085 NM Horn, The Netherlands

**Keywords:** functional status, COPD, pulmonary rehabilitation, responder analysis

## Abstract

The 6 min walking test (6MWT) has been largely studied. Less is, however, known about responders and non-responders to pulmonary rehabilitation (PR) in other meaningful activities. We explored responders and non-responders and the predictors of response to PR in the 1 min sit-to-stand test (1 min STS) and the 6MWT and compared both measures in classifying responders. An observational study was conducted with 121 people with chronic obstructive pulmonary disease (COPD). The functional status was assessed before and after PR. Baseline differences between responders and non-responders were tested with Mann–Whitney U, chi-square, or Fisher exact tests. Predictors were explored with binary logistic regressions. Agreement between both measures was assessed with chi-square, Cohen’s kappa, and McNemar tests. There were 54.5% and 57.0% of responders in the 1 min STS and the 6MWT, respectively. The proportion of responders was significantly different (*p* = 0.048), with a small agreement between the measures (kappa = 0.180; *p* = 0.048). The baseline 6MWT was the only significant predictor of response in the 6MWT (OR = 0.995; pseudo-r2 = 0.117; *p* < 0.001). No significant predictors were found for the 1 min STS. A large number of non-responders in terms of functional status exist. The 1 min STS and the 6MWT should not be used interchangeably. Future studies should explore the added benefit of personalizing PR to this outcome and investigate other potential predictors.

## 1. Introduction

Pulmonary rehabilitation (PR) is a cornerstone for the daily management of people with chronic obstructive pulmonary disease (COPD) [1]. Improvements in physical, psychological, and social traits have been widely demonstrated with this comprehensive intervention [2]. However, there are still poor responders to PR and this is partially influenced by the outcomes and measures selected [3].

Functional status, the individual’s ability to perform normal daily activities required to meet basic needs, fulfill usual roles, and maintain health and well-being [4], is a highly valued outcome of PR by patients, informal caregivers, and healthcare professionals [5]. The 6 min walking test (6MWT) is a widely used measure in PR [6], but its use is limited to long corridors and thus it cannot be applied in all settings, such as patients’ homes. Furthermore, PR should be focused on improving not only people’s ability to walk but also other meaningful activities of daily living (ADL), such as sitting down on and standing up from a chair. The 1 min sit-to-stand test (1 min STS) has been much less used but has gained popularity in recent years as it is a simple, reliable, and responsive test that elicits physiological responses similar to those by the 6MWT [7,8,9,10]. Although a responder analysis for the 6MWT has been previously conducted [3,11,12,13,14,15], less is known about the responders in the 1 min STS and whether the type of response is similar in both measures.

Hence, this study aimed to explore the (1) responders and non-responders of PR in the 1 min STS and the 6MWT, (2) predictors of response to PR in these measures, and (3) agreement between both measures in classifying responders and non-responders to PR.

## 2. Materials and Methods

### 2.1. Study Design and Population

An observational study was conducted with data collected between 2017 and 2020. The study was approved by the Ethics Committee of Centro Hospitalar do Baixo Vouga (ref. 086892), and informed consent was obtained by all participants. The study was reported following the Strengthening the Reporting of Observational Studies in Epidemiology (STROBE) statement [16].

People with a spirometry-based diagnosis of COPD (forced expiratory volume in 1 s (FEV_1_)/forced vital capacity (FVC) < 70) [17] who completed a community-based PR program were included. Those who had a history of an acute cardiac/respiratory condition within the previous month; significant cardiac, musculoskeletal, or neuromuscular diseases that impaired the ability to perform tests; signs of cognitive impairment; and a history of neoplasia or immunological disease were excluded. A complete case analysis was performed, with only variables with less than 5% missing data considered for the analysis [18]. 

### 2.2. Measurements

Sociodemographic and anthropometric data, such as age, sex, height, weight, and body mass index, were firstly collected. Clinical data collected included data on smoking status, use of long-term oxygen therapy, use of non-invasive ventilation, comorbidities using the Charlson comorbidity index [19], severity of airflow limitation and symptom burden and risk of exacerbation as defined by the Global Initiative for Chronic Obstructive Lung Disease (GOLD) [20], lung function with spirometry, respiratory-related hospital admissions, acute exacerbations of COPD, impact of the disease with the COPD assessment test (CAT) [21], dyspnea during activities with the modified medical research council dyspnea scale (mMRC) [22], health-related quality of life with Saint George’s respiratory questionnaire (SGRQ) [23], quadriceps maximum voluntary contraction with a handheld dynamometer [24] (microFET2, Hoggan Health, The best Salt Lake City, Utah), handgrip strength with a hand dynamometer (W50174, Baseline, UK), physical activity with the brief physical activity assessment tool (BPAAT) [25], balance with the brief balance evaluation systems test (Brief-BESTest) [26], and functional status with the 6MWT [27] and the 1 min STS [28]. Measurements were taken at baseline and after PR.

Participants with impairment in the 6MWT and the 1 min STS were defined as those with values below 70% of the percentage predicted [29]. For the 6MWT, the percentage predicted was computed from the equation proposed by Marques and colleagues [30] and for the 1 min STS, the reference values established by Strassmann and colleagues were used [28]. Responders were defined based on previously established minimal clinically important differences. For the 6MWT, responders were those with a pre–post mean difference of 30 m or more and non-responders were those with a change of less than 30 m [6]. For the 1 min STS, responders were those with a mean difference of 3 or more repetitions and non-responders were those with a mean difference of less than 3 repetitions [7]. 

### 2.3. Intervention

Participants completed a 12-week community-based PR program, with exercise training (aerobic and resistance muscle strength training) twice a week and education and psychosocial support once every 2 weeks. The program was provided by a multidisciplinary team of physiotherapists, medical doctors, nurses, psychologists, dietitians, and social workers. Details of the program have been published elsewhere [31].

### 2.4. Data Analysis

Descriptive statistics were computed for baseline characteristics. Effects of PR were explored through paired-samples *t*-test, Wilcoxon, and Chi-square tests as appropriate, and effect sizes were computed using Cohen’s d estimates.

Baseline differences between responders and non-responders were analyzed through independent samples *t*-tests, Mann–Whitney U tests, chi-square, or Fisher exact tests depending on data distribution. Normal distributed variables were reported as the mean ± standard deviation, non-normal distributed variables as the median (interquartile range), and frequencies as *n* (%).

Possible relationships between the mean difference in functional status and other outcomes were explored with Spearman correlations. Potential predictors of good response were explored with binary logistic regressions using a forward conditional model. Correlations were interpreted as follows: <30 small, 0.30–0.49 medium, and ≥0.50 large [32].

Chi-square tests were performed to compare the proportion of responders and non-responders in the 6MWT and in the 1 min STS. The agreement between the two measures in classifying responders and non-responders was assessed using Cohen’s kappa and McNemar tests. Cohen’s kappa was interpreted as ≤0 indicating no agreement, 0.01–0.20 none-to-slight agreement, 0.21–0.40 fair agreement, 0.41–0.60 moderate agreement, 0.61–0.80 substantial agreement, and 0.81–1.00 almost perfect agreement [33].

All analysis were performed using SPSS Statistics (v27, IBM). Plots were created using Prism (v7, GraphPad Software).

## 3. Results

### 3.1. Sample Characteristics

One hundred and twenty-one individuals were included. Most participants were male (81.8%), with a median FEV_1_ of 50% predicted, mostly GOLD grades 2 (43.0%) and 3 (38.8%) and GOLD group B (54.5%). At baseline, 48.8% of participants had an impairment in the 1 min STS and 22.3% in the 6MWT. The full baseline characteristics of participants are presented in Table 1.

### 3.2. General Effects of PR

PR was effective in improving all outcomes (*p* < 0.05) excepting BMI (*p* = 0.591) and handgrip strength (*p* = 0.619) (Table 1). Improvements above the minimal clinically important difference were seen in the number of repetitions on the 1 min STS (median_diff_ 3.0; ES = 0.58; *p* < 0.001) and on the distance walked in the 6MWT (median_diff_ 41.0; ES = 0.56; *p* < 0.001).

Negative and small-to-moderate correlations were found between the mean change in the 1 min STS and the mean change in the mMRC (r_s_ = −0.249; 95% CI (−0.415; −0.068); *p* = 0.006) and the SGRQ (r_s_ = −0.279; 95% CI (−0.441; −0.099); *p* = 0.002). A positive and moderate correlation was found between the mean change of the 1 min STS and the mean change of the 6MWT (r_s_ = 0.317; 95% CI (0.141; 0.473); *p* < 0.001).

Small correlations were also found between the mean change in the 6MWT and the mean change in the SGRQ (r_s_ = −0.197; 95% CI (−0.368; −0.013); *p* = 0.031) and the handgrip strength (r_s_ = 0.193; 95% CI (0.010; 0.364); *p* = 0.034). No other correlations were found (Tables S1 and S2 of Supplementary Materials).

### 3.3. Responders, Non-Responders, and Predictors of Response

After PR, 54.5% of the patients were responders in the 1 min STS and 57% in the 6MWT. Differences between the baseline characteristics of responders and non-responders in both the 1 min STS and the 6MWT were found. Responders in the 1 min STS had a higher baseline BMI (27.0 (24.3–30.1)) kg/m^2^ vs. non-responders (24.2 (22.1–28.3)) kg/m^2^ (*p* = 0.008) and a lower baseline performance (22.5 (18.0–27.0)) repetitions vs. non-responders (25.0 (20.5–31.0)) repetitions (*p* = 0.035). Responders in the 6MWT had lower BPAAT scores (0.0 (0.0–2.0)) points vs. non-responders (1.0 (0.0–4.0)) points (*p* = 0.038), walked a smaller distance (390.0 (295.0–480.0)) m vs. non-responders (489.2 (363.2–534.5)) m (*p* < 0.001), and had a higher percentage of people with a functional capacity impairment (30.4%) vs. non-responders (11.5%; *p* = 0.013).

When comparing the subgroups of responders and non-responders of both tests (*n* = 43, 35.5% vs. *n* = 78, 64.5%), differences were found, with responders having a higher BMI (27.5 (25.0–30.2)) kg/m^2^ vs. non-responders (24.6 (22.4–28.6)) kg/m^2^ (*p* = 0.006), showing worse performance in the 1 min STS (22.0 (17.5–26.5) repetitions vs. non-responders (24.5 (20.0–30.0)) repetitions (*p* = 0.041), being more frequently impaired in the 1 min STS (65.1%) vs. non-responders (39.7%; *p* = 0.008), walking a smaller distance in the 6MWT (386.4 (287.4–478.1)) m vs. non-responders (441.3 (356.2–523.5)) m (*p* = 0.042), and being more frequently impaired in the 6MWT (34.5%) vs. non-responders (15.4%; *p* = 0.014). No other significant differences were found.

Detailed comparisons of the baseline characteristics of responders and non-responders can be found in Table 2.

A positive and moderate correlation was found between the mean difference in the 1 min STS and the baseline BMI (r_s_ = 0.32; 95% CI (0.143; 0.474); *p* < 0.001). Negative and low correlations were also found between the mean difference in the 1 min STS and the baseline GOLD grade (r_s_ = −0.22; 95% CI (−0.390; −0.040); *p* = 0.014) and the 1 min STS (r_s_ = −0.20; 95% CI (−0.368; −0.014); *p* = 0.030) (Table S3 Supplementary Materials). 

Negative and moderate and low correlations were found between the mean difference in the 6MWT and the baseline 6MWT (r_s_ = −0.33; 95% CI (−0.483; −0.154); *p* < 0.001) and the baseline 1 min STS (r_s_ = −0.19; 95% CI (−0.360; −0.005); *p* = 0.038). No other significant correlations were found (Table S4 Supplementary Materials).

No significant predictors were found for being a good responder in the 1 min STS (*p* = 0.05) (Table S5 Supplementary Materials). The baseline 6MWD was the only significant predictor of a response to PR in the 6MWT (*p* = 0.002), with decreases of 1 m in the baseline 6MWT corresponding to 1.0 increasing the odds of being a good responder (OR = 0.995 95% CI (0.992; 0.998); pseudo-r2 = 0.117; *p* < 0.001) (Table S6 Supplementary Materials).

### 3.4. Comparison of Responders and Non-Responders Classified with the 1 min STS and the 6MWT

There were significant differences in the proportion of responders and non-responders in the two measures (*p* = 0.048), with 35.5% being responders in both measures, 24.0% being non-responders in both measures, 19.0% being responders only in the 1 min STS, and 21.5% being responders only in the 6MWT. 

**Figure 1:**
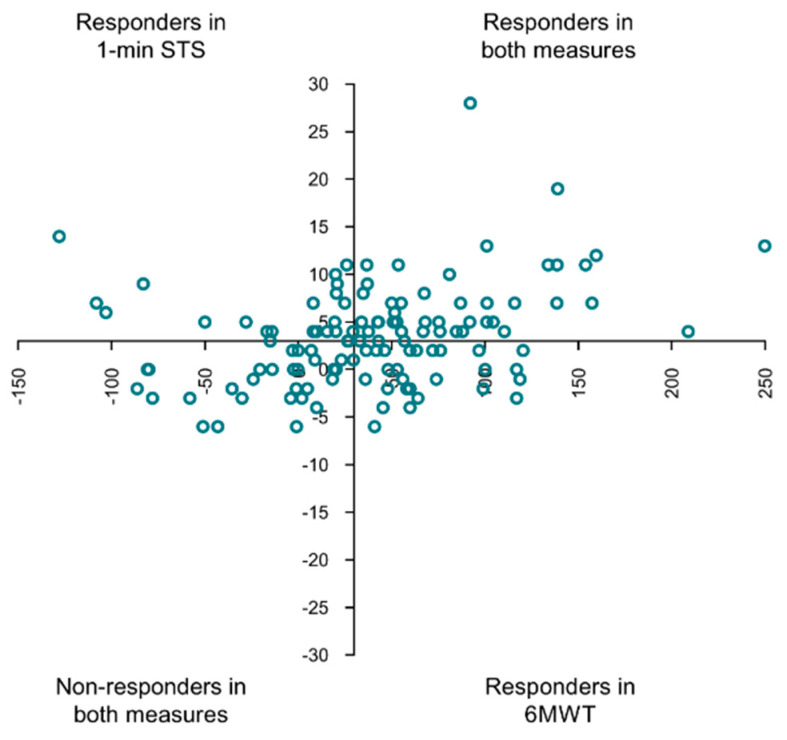
Scatter plot of responders and non-responders to community-based pulmonary rehabilitation of people with chronic obstructive pulmonary disease (COPD) with the mean difference achieved in the 6 min walking test (*X*-axis) and the 1 -min sit-to-stand test (*Y*-axis). The axes are intersected at minimal clinically important differences, 6MWT: 30 m and 1 min STS: 3 repetitions. 1 min STS: 1 min sit-to-stand test; 6MWT: 6 min walking test.

There was a slight but significant agreement between the two measures (kappa = 0.180; p_kappa_ = 0.048; p_McNemar_ = 0.755) in classifying responders and non-responders. Individual distributions of mean differences achieved in both tests across responders and non-responders can be seen in Figure 1.

## 4. Discussion

Our study has shown that although PR is effective in improving the functional status, there are also a significant number of non-responders in this outcome, independently of the measure used. This result is coherent with other studies that have shown a large and similar proportion of non-responders in the 6MWT after PR [3,11,34,35]. Since functional status is a highly valued outcome [5,36] and one of the goals of PR is to improve the physical condition, it is important to better understand why some patients are not responding to the intervention in several activities of daily living and how to better tailor PR to these patients.

Including ADL-specific training in PR has been shown to further improve the impact of the disease [37]; the average oxygen uptake; and the time to perform ADL activities, such as stair climbing, which were correlated with improvements in the 6MWT [38]. Nonetheless, the authors did not perform a responder analysis and, therefore, the impact of such intervention on the proportion of responders is unknown. It is, therefore, possible that the addition of specific ADL training to the standard aerobic and resistance training is needed, at least for some people, to achieve additional benefits in terms of not only functional capacity but also functional performance and, hence, optimize each person’s functional status. Furthermore, since non-responders seem to be more fit at baseline than those responding to PR, it is possible that these measures are not responsive enough for these patients or that more challenging exercise modalities, such as high-intensity functional training [39], are needed. The best way to tailor PR to this outcome is, however, yet to be determined. 

Additionally, these measures might not be sufficient to have a comprehensive view of the patient functional status as they are focused on functional capacity. In fact, only low-to-moderate correlations were found between both physical tests and between these tests and other important outcomes, such as health-related quality of life. Similar results have been reported during acute states of the disease [40]. Therefore, using composite measures of functional capacity and functional performance, such as the combined use of physical tests and patient-reported outcome measures, might be the way forward to provide a full picture of the patient functional status.

Patients with higher baseline BMIs, worse physical activity levels, and worse performance in the 1 min STS and the 6MWT seem to be better responders in terms of functional status. Having a worse baseline status and being a good responder is a commonly observed pattern for several outcomes, and it might be due to having more room for improvement [3,35,41]. A low baseline 6MWD was the only significant predictor of good response, with little explanation for the variability in the response. Hence, reasons for being a good or poor responder in this outcome remain unclear and require further investigation.

Other patient features, such as muscle function or body composition, could potentially explain why patients respond or do not respond to the intervention. A previous study has shown isometric quadriceps muscle strength to be a predictor of response to PR in the 6MWT [42]. However, we did not find it as a predictor and a recent study found no correlations between isometric measurements of muscle function and the 1 min STS, with muscle power measured by isokinetic or the 5-time STS being the main contributor to the 1 min STS and the 6MWT performance [43]. Hence, dynamic measurements and more robust measures (e.g., isokinetic) should be further explored in the future, as they could be highly relevant to assess and predict the response to PR.

Responders and non-responders in each test shared some features at baseline, such as a similar age, severity of airflow obstruction, comorbidities, symptoms, muscle strength, health-related quality of life, and balance. Thus, improvements in functional status seem to occur independently of these characteristics, and therefore they should prevent healthcare professionals from planning exercise interventions to target this outcome.

We found significant differences in the proportion of responders between the 1 min STS and the 6MWT, with a small agreement between the two measures in classifying responders and non-responders. This result suggests that using the 1 min STS and the 6MWT interchangeably might not be the most appropriate approach, as it can lead to a misclassification of the person as a good or poor responder to PR in this outcome. In fact, since both tests mimic different ADL, it is possible that some people find it more difficult to stand up from a chair repeatedly than to perform a self-paced walk and vice-versa. Although similar physiological responses (e.g., oxygen consumption) have been found between both measures [7], other factors, such as muscle fatigue or mobility, may play a role in explaining the differences in response to the two measures. Differences due to the different contributions of muscle strength, power, and endurance to the 1 min STS and the 6MWT might explain differences obtained [42], but this was not tested in this study. Caution is, therefore, recommended when using the 1 min STS as a surrogate measure of the 6MWT.

This study has some limitations. Although we explored correlations between the type of response with commonly used measures, other measures, such as DEXA and isokinetic muscle assessment, could have proved stronger predictors of response to PR in terms of functional status. Additionally, other patient characteristics, such as fatigue, objective physical activity, and mobility, may influence the response to PR in this outcome and should, therefore, be explored in future studies. 

## 5. Conclusions

Community-based pulmonary rehabilitation improves the functional status of people with COPD; however, a large number of non-responders exist. A low baseline 6MWD was the only significant predictor of a good response in the 6MWT, with no predictors found for the 1 min STS. Future studies should explore the added benefit of tailoring PR to this outcome (e.g., including ADL training) to maximize the response to PR. The small agreement in classifying responders and non-responders between the 1 min STS and the 6MWT suggests that these measures should not be used interchangeably to assess the results of PR in this outcome. Future prospective studies with larger samples are needed to confirm these findings and explore other potential factors influencing the response to PR in this outcome.

## Figures and Tables

**Table 1:** Baseline characteristics and effects of pulmonary rehabilitation in people with chronic obstructive pulmonary disease (COPD) (*n* = 121).

	Baseline	Post	∆ Pre-Post	Effect Size (d)	*p*-Value
Age, years	69.0 (65.0–75.0)				
Sex, *n* (%)					
Male	99 (81.8)				
Female	22 (18.2)				
BMI, kg/m^2^	26.4 ± 4.8	26.4 ± 4.6	−0.0 ± 0.9	0.049	0.591
Underweight, <21, *n* (%)	13 (10.7)	10 (8.3)	3 (2.4)		<0.001 *
Obese >30, *n* (%)	28 (23.1)	27 (22.5)	1 (0.6)		
Smoking status, *n* (%)					
Never	22 (18.2)				
Former	82 (67.8)				
Current	17 (14.0)				
Pack-years	31.2 (10.0–60.0)				
LTOT, *n* (%)	12 (9.9)				
NIV, *n* (%)	16 (13.2)				
CCI, score	4.0 (3.0–5.0)				
FEV_1_ % predicted	50.0 (37.0–63.7)				
GOLD grade, *n* (%)					
1	9 (7.4)				
2	52 (43.0)				
3	47 (38.8)				
4	13 (10.7)				
GOLD group, *n* (%)					
A	32 (26.4)				
B	66 (54.5)				
C	3 (2.5)				
D	20 (16.5)				
Respiratory-related hospital admissions in the previous 12 months, *n*	0.0 (0.0–0.0)				
AECOPD in the previous 12 months, *n*	0.0 (0.0–1.0)				
mMRC, score	2.0 (1.0–3.0)	1.0 (1.0–2.0)	0.0 (−1.0–0.0)	0.39	<0.001 *
CAT score, points	14.8 ± 8.0	12.1 ± 7.0	−2.7 ± 5.8	0.46	<0.001 *
SGRQ, total score	45.1 ± 19.8	38.7 ± 19.1	−6.4 ± 12.2	0.52	<0.001 *
QVC, KgF	30.6 ± 8.4	33.4 ± 10.2	2.7 ± 8.7	0.31	<0.001 *
Handgrip strength, KgF	34.3 ± 9.2	34.0 ± 10.1	−0.3 ± 7.5	0.05	0.619
BPAAT, score	0.0 (0.0–3.0)	4.0 (2.0–6.0)	2.0 (1.0–4.0)	0.69	<0.001 *
Brief-BESTest	19.0 (15.0–22.0)	21.0 (18.0–24.0)	2.0 (0.0–4.0)	0.63	<0.001 *
1 min STS, repetitions	23.0 (18.0–29.0)	27.0 (21.0–33.0)	3.0 (0.0–6.0)	0.58	<0.001 *
1 min STS <70% predicted, *n* (%)	59 (48.8)	38 (31.4)	21 (17.4)		<0.001 *
6MWD, m	419.6 (331.9–508.6)	465.0 (386.7–540.3)	41.0 (7.0–75.3)	0.56	<0.001 *
6MWD < 70% predicted, *n* (%)	27 (22.3)	20 (16.5)	7 (5.8)		<0.001 *

BMI: body mass index; LTOT: long-term oxygen therapy; NIV: non-invasive ventilation; CCI: Charlson comorbidity index; FEV_1_: forced expiratory volume in the first second; GOLD: global initiative for chronic obstructive lung disease. 1–4: Severity of airflow limitation, 1—FEV_1_ ≥ 80% predicted, 2—50% ≤ FEV_1_ < 80% predicted, 3—30% ≤ FEV_1_ < 50% predicted, and 4—FEV_1_ < 30% predicted. CAT: COPD assessment test; A–D: A—CAT < 10 points and 0–1 moderate-to-severe exacerbations (not leading to hospitalization), B—CAT ≥ 10 points and 0–1 moderate-to-severe exacerbations (not leading to hospitalization), C—CAT < 10 points and ≥2 moderate-to-severe exacerbations or ≥1 moderate-to-severe exacerbations leading to hospitalization, and D—CAT ≥ 10 points and ≥2 moderate-to-severe exacerbations or ≥1 moderate-to-severe exacerbations leading to hospitalization; AECOPD: acute exacerbations of COPD; mMRC: modified medical research council dyspnea scale; SGRQ: Saint George’s respiratory questionnaire; QVC: quadriceps voluntary contraction; BPAAT: brief physical activity assessment tool; Brief-BESTest: brief-balance evaluation system test; 1 min STS: 1 min sit-to-stand test; 6MWD: 6 min walking distance. ∆ represents the mean or median difference according to data distribution. * Statistically significant difference.

**Table 2:** Baseline differences between responders and non-responders in terms of pulmonary rehabilitation of people with chronic obstructive pulmonary disease (COPD) in the 1 min sit-to-stand test and the 6 min walking test (*n* = 121).

	1-Min STS			6MWT			1-Min STS and 6MWT		
	Responders (*n* = 66)	Non-Responders (*n* = 55)	*p*-Value	Responders (*n* = 69)	Non-Responders (*n* = 52)	*p*-Value	Responders in Both Measures (*n* = 43)	Non-Responders in Both Measures (*n* = 78)	*p*-Value
Age, years	69.0 (64.0–75.0)	70.0 (67.5–74.5)	0.150	69.0 (65.0–71.5)	69.0 (65.0–71.5)	0.344	69.0 (61.5–73.0)	70.0 (66.0–75.0)	0.178
Sex, *n* (%)									
Male	50 (75.8)	49 (89.1)	0.058	57 (82.6)	42 (80.8)	0.795	34 (79.1)	65 (83.3)	0.561
Female	16 (24.2)	6 (10.9)		12 (17.4)	10 (19.2)		9 (20.9)	13 (16.7)	
BMI, kg/m^2^	27.0 (24.3–30.1)	24.2 (22.1–28.3)	0.008	27.0 ± 4.6	25.7 ± 5.0	0.143	27.5 (25.0–30.2)	24.6 (22.4–28.6)	0.009
Smoking status, *n* (%)									
Never	13 (19.7)	9 (16.4)	0.475	11 (15.9)	11 (21.2)	0.668	6 (14.0)	16 (20.5)	0.504
Former	46 (69.7)	36 (65.5)		49 (71.0)	33 (63.5)		32 (74.4)	50 (64.1)	
Current	7 (10.6)	10 (18.2)		9 (13.0)	8 (15.4)		5 (11.6)	12 (15.4)	
Pack-years, *n*	38.8 (12.3–64.0)	30.0 (6.1–55.0)	0.342	34.1 (13.1–60.0)	28.5 (3.2–69−0)	0.756	38.8 (17.0–60.0)	30.0 (5.9–60.8)	0.510
LTOT, *n* (%)	8 (12.1)	4 (7.3)	0.374	6 (8.7)	6 (11.5)	0.605	4 (9.3)	8 (10.3)	0.867
NIV, *n* (%)	11 (16.7)	5 (9.1)	0.221	11 (15.9)	5 (9.6)	0.309	9 (20.9)	7 (9.0)	0.063
CCI, score	4.0 (3.0–5.0)	4.0 (3.0–5.0)	0.513	4.0 (3.0–5.0)	4.0 (3.0–4.5)	0.313	4.0 (3.0–5.0)	4.0 (3.0–5.0)	0.980
FEV1, % predicted	54.3 ± 17.0	47.8 ± 18.4	0.049	50.4 ± 16.0	52.6 ± 20.3	0.521	54.0 (41.0–63.0)	47.0 (36.0–63.7)	0.273
GOLD grade, *n* (%)									
1	6 (9.1)	3 (5.5)	0.054	3 (4.3)	6 (11.5)	0.180	2 (4.7)	7 (9.0)	0.064
2	33 (50.0)	19 (34.5)		33 (47.8)	19 (36.5)		25 (58.1)	27 (34.6)	
3	24 (36.4)	23 (41.8)		28 (40.6)	19 (36.5)		14 (32.6)	33 (42.3)	
4	3 (4.5)	10 (18.2)		5 (7.2)	8 (15.4)		2 (4.7)	11 (14.1)	
GOLD group, *n* (%)									
A	14 (21.2)	18 (32.7)	0.525	16 (23.2)	16 (30.8)	0.405	9 (20.9)	23 (29.5)	0.375
B	39 (59.1)	27 (49.1)		42 (60.9)	24 (46.2)		28 (65.1)	38 (48.7)	
C	2 (3.0)	1 (1.8)		1 (1.4)	2 (3.8)		1 (2.3)	2 (2.6)	
D	11 (16.7)	9 (16.4)		10 (14.5)	10 (19.2)		5 (11.6)	15 (19.2)	
Respiratory-related hospital admissions in the previous 12 months, *n*	0.0 (0.0–0.0)	0.0 (0.0–0.0)	0.775	0.0 (0.0–0.0)	0.0 (0.0–0.0)	0.298	0.0 (0.0–0.0)	0.0 (0.0–0.0)	0.455
AECOPD in the previous 12 months, *n*	0.0 (0.0–1.0)	0.0 (0.0–1.0)	0.083	0.0 (0.0–1.0)	0.0 (0.0–1.0)	0.983	0.0 (0.0–1.0)	0.0 (0.0–1.0)	0.399
mMRC, score	2.0 (1.0–3.0)	2.0 (1.0–3.0)	0.441	2.0 (1.0–3.0)	2.0 (1.0–3.0)	0.679	2.0 (1.0–2.5)	2.0 (1.0–3.0)	0.420
CAT, total score	15.9 ± 8.2	13.4 ± 7.5	0.078	15.5 ± 7.9	13.8 ± 8.0	0.228	13.2 ± 7.8	14.4 ± 8.0	0.425
SGRQ, total score	53.7 (30.2–62.8)	43.1 (30.1–53.5)	0.060	52.6 (30.1–61.8)	45.1 (30.2–56.9)	0.321	53.7 (29.6–61.1)	45.1 (30.2–57.4)	0.399
QVC, KgF	30.2 ± 8.6	31.0 ± 8.2	0.561	30.7 ± 8.9	30.4 ± 7.7	0.875	30.1 ± 9.1	30.8 ± 8.0	0.679
Handgrip strength, KgF	34.3 ± 10.4	34.4 ± 7.7	0.924	34.3 ± 9.9	34.3 ± 8.4	0.987	34.6 ± 10.5	34.2 ± 8.5	0.803
BPAAT, score	0.0 (0.0–3.0)	1.0 (0.0–3.0)	0.513	0.0 (0.0–2.0)	1.0 (0.0–4.0)	0.038	0.0 (0.0–1.0)	0.5 (0.0–3.0)	0.156
Brief-BESTest, score	19.0 (14.0–22.0)	18.0 (16.0–22.0)	0.876	18.0 (15.0–22.0)	19.0 (16.0–22.0)	0.373	19.0 (13.5–22.0)	18.0 (16.0–22.0)	0.580
1 min STS, repetitions	22.5 (18.0–27.0)	25.0 (20.5–31.0)	0.035	22.0 (18.0–28.0)	25.5 (20.0–31.5)	0.113	22.0 (17.5–26.5)	24.5 (20.0–30.0)	0.041
1 min STS <70% predicted, *n* (%)	37 (56.1)	22 (40.0)	0.078	37 (53.6)	22 (42.3)	0.218	28 (65.1)	31 (39.7)	0.008
6MWD, m	404.4 ± 135.2	419.2 ± 115.9	0.524	390.0 (295.0–480.0)	489.2 (363.2–534.5)	<0.001	386.4 (287.4–478.1)	441.3 (356.2–523.5)	0.042
6MWD <70% predicted, *n* (%)	17 (25.8)	10 (18.2)	0.319	21 (30.4)	6 (11.5)	0.013	15 (34.9)	12 (15.4)	0.014

BMI: body mass index; LTOT: long-term oxygen therapy; NIV: non-invasive ventilation; CCI: Charlson comorbidity index; FEV1: forced expiratory volume in 1 s; GOLD: global initiative for chronic obstructive lung disease. 1–4: Severity of airflow limitation, 1—FEV1 ≥ 80% predicted, 2—50% ≤ FEV1 < 80% predicted, 3—30% ≤ FEV1 < 50% predicted, and 4—FEV1 < 30% predicted. CAT: COPD assessment test; A–D: A—CAT < 10 points and 0–1 moderate-to-severe exacerbations (not leading to hospitalization), B—CAT ≥ 10 points and 0–1 moderate-to-severe exacerbations (not leading to hospitalization), C—CAT < 10 points and ≥2 moderate-to-severe exacerbations or ≥1 moderate-to-severe exacerbations leading to hospitalization, and D—CAT ≥ 10 points and ≥2 moderate-to-severe exacerbations or ≥1 moderate-to-severe exacerbations leading to hospitalization; AECOPD: acute exacerbations of COPD; mMRC: modified medical research council dyspnea scale; SGRQ: Saint George’s respiratory questionnaire; QVC: quadriceps voluntary contraction; BPAAT: brief physical activity assessment tool; Brief-BESTest: brief-balance evaluation systems test; 1 min STS: 1 min sit-to-stand test; 6MWD: 6 min walking distance.

## Data Availability

The data presented in this study are available from the corresponding author on reasonable request.

## Supplementary Materials

The following supporting information can be downloaded at: www.mdpi.com/article/10.3390/jcm11030518/s1, Table S1: Correlation coefficients between mean difference in 1 min sit-to-stand test and mean differences of all variables included in analysis; Table S2: Correlation coefficients between mean difference in 6 min walk test and mean differences of all variables included in analysis; Table S3: Correlation coefficients between mean difference in 1 min sit-to-stand test and baseline characteristics; Table S4: Correlation coefficients between mean difference in 6 min walk test and baseline characteristics; Table S5: Logistic regression for response in 1 min STS; Table S6: Logistic regression for response in 6MWT.
