# Supplementary material for: Functional Status Following Pulmonary Rehabilitation: Responders and Non-Responders
Source: J Clin Med. 2022 Jan 20;11(3):518. doi: 10.3390/jcm11030518 (PMC8836346; doi:10.3390/jcm11030518)
Supplement: Supplementary file 1 [file jcm-11-00518-s001.zip › jcm-1495808-supplementary.pdf]

Functional status following pulmonary rehabilitation: responders and non-responders

Sara Souto-Miranda[1,2,3], Maria A. Mendes[4], João Cravo[4], Lília Andrade[4], Martijn A. Spruit[3,5], Alda Marques[1]

[1]Lab3R - Respiratory Research and Rehabilitation Laboratory, School of Health Sciences (ESSUA) and Institute of Biomedicine (iBiMED), University of Aveiro, Portugal;

[2]Department of Medical Sciences, University of Aveiro, Aveiro, Portugal;

[3]Department of Respiratory Medicine, Maastricht University Medical Centre, NUTRIM School of Nutrition and Translational Research in Metabolism, Faculty of Health, Medicine and Life Sciences, Maastricht University, The Netherlands;

[d]Department of Pulmonology, Centro Hospitalar do Baixo Vouga, E.P.E.;

[e]Department of Research and Development, CIRO, Horn, The Netherlands.

Corresponding author: Alda Marques, Respiratory Research and Rehabilitation Laboratory (Lab3R), School of Health Sciences (ESSUA) and Institute of Biomedicine (iBiMED), University of Aveiro, Agras do Crasto - Campus Universitário de Santiago, Edifício 30, 3810-193 Aveiro, Portugal. Tel +351234372462. Email amarques@ua.pt

Table S1. Correlation coefficients between mean difference in 1-minute sit-to-stand test and mean differences of all variables included in analysis.

BMI: Body mass index; mMRC: Modified medical research council dyspnoea scale; BPAAT: Brief physical activity assessment tool; CAT: COPD assessment test; SGRQ: Saint George's respiratory questionnaire; 6MWT: Six-minute walk test; QVC: Quadriceps voluntary contraction; Brief-BESTest: Brief-Balance evaluation systems test.

**Variable**	**Correlation coefficient**	**95% CI**	**p-value**
BMI	-0.097	0.294;-0.276	0.294
mMRC	-0.249	-0.415;-0.068	**0.006**
BPAAT	-0.148	-0.324;0.037	0.106
CAT	-0.118	-0.295;0.067	0.197
SGRQ	-0.279	-0.441;-0.099	**0.002**
6MWT	0.317	0.141;0.473	**<0.001**
QVC	0.071	-0.115;0.252	0.441
Handgrip strength	0.096	-0.089;0.275	0.295
Brief-BESTest	0.023	-0.167;0.212	0.806

Table S2. Correlation coefficients between mean difference in 6-minute walk test and mean differences of all variables included in analysis.

**Variable**	**Correlation coefficient**	**95% CI**	**p-value**
BMI	-0.075	-0.256;0.111	0.418
mMRC	-0.158	-0.333;0.027	0.084
BPAAT	0.152	-0.034;0.327	0.098
CAT	-0.025	-0.208;0.159	0.783
SGRQ	-0.197	-0.368;-0.013	**0.031**
QVC	0.059	-0.127;0.241	0.524
Handgrip strength	0.193	0.010;0.364	**0.034**
1-min STS	0.317	0.141;0.473	**<0.001**
Brief-BESTest	0.099	-0.092;0.283	0.294

BMI: Body mass index; mMRC: Modified medical research council dyspnoea scale; BPAAT: Brief physical activity assessment tool; CAT: COPD assessment test; SGRQ: Saint George's respiratory questionnaire; 1-min STS: 1-minute sit-to-stand test; QVC: Quadriceps voluntary contraction; Brief-BESTest: Brief-Balance evaluation systems test.

Table S3. Correlation coefficients between mean difference in 1-minute sit-to-stand test and baseline characteristics.

**Variable**	**Correlation coefficient**	**95% CI**	**p-value**
Sex	0.148	-0.037;323	0.106
Age	-0.060	-0.241;0.126	0.517
Smoking status	-0.138	-0.314;0.047	0.130
Pack-years	0.047	-0.140;0.231	0.612
CCI_Baseline	-0.022	-0.204;0.163	0.814
BMI	0.318	0.143;0.474	**<0.001**
LTOT	-0.039	-0.221;0.146	0.672
NIV	0.074	-0.112;0.254	0.422
Hospital admissions previous 12 months	-0.038	-0.220;0.147	0.681
AECOPD previous 12 months	0.144	-0.041;0.319	0.116
mMRC	0.062	-0.123;0.243	0.500
BPAAT	.073	-0.112;0.254	0.423

CAT	0.118	-0.067;0.295	0.198
GOLD group (A-D)	0.042	-0.143;0.224	0.647
SGRQ	0.129	-0.056;0.307	0.159
FEV1, % predicted	0.164	-0.021;0.337	0.073
GOLD grade (1-4)	-0.222	-0.390;-0.040	**0.014**
6MWT	0.011	-0.173;0.195	0.902
QVC	0.078	-0.107;0.258	0.393
1-min STS	-0.198	-0.368;-0.014	**0.030**

CCI: Charlson comorbidity index; BMI: Body mass index; LTOT: Long-term oxygen therapy; NIV: Non-invasive ventilation; mMRC: Modified medical research council dyspnoea scale; BPAAT: Brief physical activity assessment tool; CAT: COPD assessment test; GOLD: Global initiative for chronic lung disease; SGRQ: Saint George's respiratory questionnaire; FEV1: Forced expiratory volume in 1 second; 6MWT: Six-minute walk test; QVC: Quadriceps voluntary contraction; 1-min STS: 1-minute sit-to-stand test.

Table S4. Correlation coefficients between mean difference in 6-minute walk test and baseline characteristics.

**Variable**	**Correlation coefficient**	**95% CI**	**p-value**
Sex	0.102	-0.083;0.281	0.263
Age	0.094	-0.091;0.273	0.305
Smoking status	0.021	-0.163;0.204	0.817
Pack-years	-0.106	-0.286;0.082	0.254
CCI_Baseline	0.109	-0.076;0.287	0.234
BMI	0.114	-0.072;0.291	0.215
LTOT	-0.080	-0.260;0.105	0.381
NIV	0.000	-0.183;0.184	0.997
Hospital admissions previous 12 months	-0.032	-0.215;0.152	0.725
AECOPD previous 12 months	0.112	-0.073;0.290	0.222
mMRC	-0.010	-0.193;0.174	0.912
BPAAT	-0.109	-0.287;0.076	0.234
CAT	0.114	-0.071;0.291	0.214
GOLD group (A-D)	0.057	-0.128;0.238	0.533
SGRQ	0.091	-0.095;0.271	0.321
FEV1, % predicted	-0.009	-0.193;0.175	0.919
GOLD grade (1-4)	-0.042	-0.224;0.142	0.645
6MWT	-0.329	-0.483;-0.154	**<0.001**
QVC	-0.095	-0.274;0.090	0.300
Handgrip strength	-0.062	-0.243;0.123	0.502
1-min STS	-0.189	-0.360;-0.005	**0.038**
Brief-BESTest	-0.077	-0.260;0.113	0.414

CCI: Charlson comorbidity index; BMI: Body mass index; LTOT: Long-term oxygen therapy; NIV: Non-invasive ventilation; mMRC: Modified medical research council dyspnoea scale; BPAAT: Brief physical activity assessment tool; CAT: COPD assessment test; GOLD: Global initiative for chronic lung disease; ; SGRQ: Saint George's respiratory questionnaire; FEV1: Forced expiratory volume in 1 second; 6MWT: Six-minute walk test; QVC: Quadriceps voluntary contraction

Table S5. Logistic regression for response in 1-min STS.

**Variables in equation**	**Coefficient**	**p-value**	**Odds ratio**	**95%CI**

Baseline 1-min STS	-0.042	0.050	0.958	0.919;1.000
Constant	1.233	0.030	3.433	
**Variables not in equation**				
Baseline BMI		0.116		

1-min STS: 1-minute sit-to-stand test; BMI: Body mass index

Table S.6. Logistic regression for response in 6MWT.

Variables in equation	Coefficient	p-value	Odds ratio	95%CI
Baseline 6MWT	-0.005	0.002	0.995	0.992;0.998
Constant	2.452	<0.001	11.613	
**Variables not in equation**				
Baseline 1-min STS		0.168		

6MWT: Six-minute walk test; 1-min STS: 1-minute sit-to-stand test
